# Successful immune tolerance induction consisting of high-dose factor VIII rich in von Willebrand factor and pulsed intravenous immunoglobulin: a case report

**DOI:** 10.1186/1752-1947-6-350

**Published:** 2012-10-11

**Authors:** Peter Kubisz, Ivana Plamenova, Pavol Holly, Jan Stasko

**Affiliations:** 1 Department of Hematology and Transfusion Medicine, Jessenius Faculty of Medicine of the Comenius University and University Hospital Martin, Kollarova 2, Martin 036 59, Slovakia

**Keywords:** Coagulation factor VIII inhibitor, Hemophilia A, Immune tolerance induction, Intravenous immunoglobulin, Recombinant activated factor VII

## Abstract

**Introduction:**

The development of factor VIII inhibitors is a serious complication of replacement therapy in patients with congenital hemophilia A. Immune tolerance induction has been accepted as the only clinically proven treatment allowing antigen-specific tolerance to factor VIII. However, some of its issues, such as patient selection, timing, factor VIII dosing, use of immunosuppressive or immunomodulatory procedures, still remain the subject of debate.

**Case presentation:**

A case of a 3-year-old Caucasian boy with severe congenital hemophilia A, intron 22 inversion of the *F8* gene and high-titer inhibitor, who underwent an immune tolerance induction according to the modified Bonn regimen (high doses of plasma-derived factor VIII rich in von Willebrand factor and pulsed intravenous immunoglobulin) is presented. The treatment lasted for 13 months and led to the eradication of inhibitor.

**Conclusion:**

Addition of intravenous immunoglobulin did not negatively affect the course of immune tolerance induction and led to the rapid eradication of factor VIII inhibitor.

## Introduction

The development of factor VIII (FVIII) inhibitors is a serious complication of replacement therapy in patients with congenital hemophilia A, affecting up to 35%, predominantly those with severe disease [1]. It leads to the failure of previously efficient replacement therapy or prophylaxis with FVIII and thus to an increased risk of bleeding, joint impairment and reduced quality of life. Immune tolerance induction (ITI) has been accepted as the only clinically proven treatment allowing antigen-specific tolerance to FVIII [2,3]. However, some of its issues, such as patient selection, timing, FVIII dosing, use of immunosuppressive or immunomodulatory procedures, still remain unclear. Several modifications of the first ITI treatment proposed in Bonn (Bonn regimen) have been described, but none have been proved to be superior [3-7].

## Case presentation

We present a successful use of the high-dose FVIII regimen for ITI: plasma-derived FVIII rich in von Willebrand factor (vWF) and pulsed intravenous immunoglobulin (IVIG). The high-dose FVIII regimen was used in a 3-year-old Caucasian boy with severe congenital hemophilia A (endogenous FVIII level below 0.01IU/mL), the intron 22 inversion of the *F8* gene and high-titer FVIII inhibitor. The patient was treated with on-demand replacement therapy with a single human plasma-derived FVIII concentrate rich in vWF (Fanhdi^®^, Grifols, Barcelona, Spain) from the age of 2 months. The initial choice of FVIII concentrate was made according to the availability of FVIII products at the start of the treatment. The FVIII inhibitor in a high titer occurred after 2 years of treatment and 20 exposure days (initial inhibitor titer (IT): 5.4BU/mL; Day −453, 453 days before the start of ITI). In order to prevent further immunization, FVIII was omitted from the treatment and on-demand recombinant activated factor VII (rFVIIa; NovoSeven^®^, NovoNordisk^®^, Bagsværd, Denmark) was used instead for maintaining hemostasis. The activated prothrombin complex concentrate factor VIII inhibitor bypass activity (FEIBA [factor eight inhibitor bypass activity]; Baxter International Inc., Deerfield, IL, USA) was not preferred due to the known risk of anamnestic response and prolongation of time period necessary for IT decrease. Within 14 months IT spontaneously decreased to 0.8BU/mL and thus, at the age of 3 years and 20 days, the first ITI could be started. The previously described ITI regimen – a combination of high doses of FVIII as in the original Bonn protocol (100IU/kg intravenous (IV) twice a day; the same FVIII concentrate as in the previous treatment) and pulsed IVIG (1g/kg IV on Days 0, 1, 7, 15 and 21; Kiovig, Baxter AG, Wien, Austria) – was used [5].

**Figure 1:**
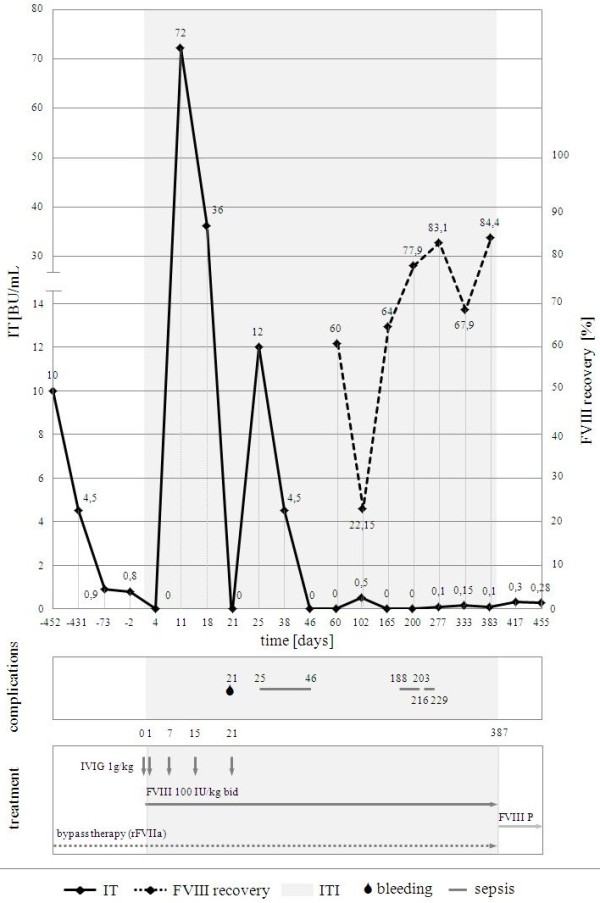
**The time changes in initial inhibitor titer (IT) and factor VIII (FVIII) recovery before and during immune tolerance induction (ITI).** IT constantly decreased after the FVIII discontinuation; during ITI, after the initial increase (up to 72.0BU/mL) in the first month (anamnestic response), a constant decrease in IT was seen with a transient low increase (up to 12.0BU/mL) during severe infection (catheter-related sepsis) in the second month; FVIII recovery continuously improved from the fourth month of ITI. BU = Bethesda unit; FVIII = factor VIII; FVIII P = prophylaxis with factor VIII; IT = inhibitor titer; ITI = immune tolerance induction; IU = international unit; rFVIIa = recombinant activated factor VII.

Invasive procedures as well as bleedings were managed with on-demand rFVIIa (90μg/kg IV as a bolus, repeated every 2 hours until the cessation of bleeding). The stable IV access was secured via the central venous access device (CVAD; PORT-A-CATH^^®^^, Smiths Medical, St. Paul, MN, USA) placed in the subclavian vein. IT immediately before the start of ITI (Day −3, 3 days before the start of ITI) was 0.8BU/mL; its highest level was 10.0BU/mL. During ITI, the highest IT was observed in the first month (72.0BU/mL), whereas from the second month only low IT was found with a transient increase during infections. After 12 months IT decreased to 0.1BU/mL and the FVIII pharmacokinetics normalized (recovery time 30 minutes after FVIII: 84.4%; plasma half-life: 6.5 hours; IV response to FVIII: 1.69IU/kg; normal FVIII clearance; testing performed on Day 383 of ITI). Thus, ITI was considered successful and terminated on Day 387 of ITI (Figure 1). No adverse events directly associated with the administered agents were observed during ITI.

Only one serious hemorrhage (intramuscular in the left thigh; Day 21 of ITI) occurred; its cessation was achieved solely with bypass therapy. However, ITI was complicated with severe bacterial infections associated with CVAD (three episodes of catheter-related sepsis; Days 25, 188, and 216 of ITI).

The prophylactic re-treatment with the same plasma-derived FVIII (Fanhdi^^®^^ 1000IU IV thrice a week without gradual tapering) was initiated after ITI termination. During a 10-month follow-up, no clinical or laboratory signs of FVIII inhibitor and no severe bleeding appeared.

## Discussion

Although current knowledge of ITI is based predominantly on results of uncontrolled retrospective studies, case reports and patient registries (International Immune Tolerance Registry, German Registry, North American Immune Tolerance Registry) with limited directed comparison of different regimens, it is commonly accepted that the outcome of ITI is worse in patients with IT more than 10BU/mL at ITI initiation, historical IT more than 200BU/mL and peak IT on ITI more than 250BU/mL [3,7]. Other negative factors – age over 20 years at beginning of ITI, persistence of inhibitor for more than 5 years, use of FVIII concentrates without vWF, presence of null mutations within *F8* gene, African origin, and inflammatory challenges during ITI – were suggested by particular analyses [7-13]. However, the available clinical data on those parameters are not consistent and sufficient for their general acceptance. The recently finished International Immune Tolerance Study, involving 115 good-risk patients with severe congenital hemophilia A and high-titer inhibitor on ITI, verified the inverse correlation of historical IT and peak IT on ITI with the treatment success, but failed to show a significant effect of infections on its outcome [14].

The positive effect of IVIG addition to ITI has been reported since the introduction of the Malmö protocol [3,15]. The rapid, although time-limited, improvement in FVIII pharmacokinetics and decrease in IT, accompanied by lower bleeding tendency, was attributed to IVIG [15]. However, these reports are anecdotal and limited almost exclusively to case reports. Therefore, a recent international consensus recommendation does not approve the standard use of IVIG or other immunomodulating agents in first-line ITI [13].

In the presented case, the patient had a good prognostic profile, with the low initial, historical, and anamnestic IT and with only one known negative factor (intron 22 inversion). The treatment was well tolerated with relatively rare bleedings. CVAD-related sepsis was regarded as the most serious complication. The infections, although recurrent and associated with the transient elevation of IT, did not result in a marked prolongation of ITI. The normalization of FVIII pharmacokinetics was seen after 13 months, so the ITI duration was comparable with the mean durations reported in the analyses of patient registries [3,7]. The use of IVIG could, similarly to the observations of other authors, contribute to the relatively low frequency of severe hemorrhages and the normal ITI duration despite repeated inflammatory challenges [4,12].

The bleeding episodes in hemophilia patients with high-titer inhibitor, both prior to and during ITI, are managed with bypass agents: rFVIIa and activated prothrombin complex concentrate FEIBA. Both agents are comparable in terms of efficacy and safety. However, the ability of FEIBA to induce anamnestic response, and thus prolong the persistence of inhibitor, is a possible drawback for its use in ITI. Although the negative effect of this phenomenon on ITI outcome is not clear, FEIBA is nowadays not recommended for the first-line treatment in patients planned for ITI [13]. In concordance with the standard practice, rFVIIa was preferred in the presented case.

## Conclusions

We report a successful use of ITI consisting of high-dose plasma-derived FVIII rich in vWF and IVIG in a hemophilia patient with high-titer FVIII inhibitor and intron 22 inversion. The addition of IVIG did not alter the course of ITI or the patient’s condition.

## Consent

Written informed consent was obtained from the patient’s legal guardian (mother) for publication of this case report and any accompanying images. A copy of the written consent is available for review by the Editor-in-Chief of this journal.

## Abbreviations

BU: Bethesda unit; CVAD: Central venous access device; FEIBA: Factor VIII inhibitor bypass activity; FVIII: Factor VIII; IT: Inhibitor titer; ITI: Immune tolerance induction; IU: International unit; IV: Intravenous; IVIG: Intravenous immunoglobulin; rFVIIa: Recombinant activated factor VII; vWF: von Willebrand factor.

## Competing interests

All authors declare that they have no competing interests regarding this work.

## Authors’ contributions

PK and IP conceived this case report and were responsible for the management of the patient. PH was a major contributor in writing the manuscript and reviewing the literature data. JS made substantial contributions to the interpretation of data and participated in revising the manuscript. All authors read and approved the final manuscript.
